# Research on measurement technology of optical fiber angle sensor based on MEA-BP

**DOI:** 10.1038/s41598-026-48008-1

**Published:** 2026-04-11

**Authors:** Wang Lisha, Zhang Jing, Wan Pu, Liu Yinxu, Yang Desheng, Li Xin, Lisha Wang

**Affiliations:** School of Physics and Information Engineering, Zhaotong University, Zhaotong, 657000 YunnanChina China

**Keywords:** High-voltage disconnector, Fiber optic sensor, Evolutionary algorithm, BP neural network, Nonlinear compensation, Engineering, Optics and photonics

## Abstract

High-voltage disconnectors operate under strong electromagnetic radiation for long periods, making it prone to incomplete opening and closing. Current electronic sensors used to detect their status are susceptible to electromagnetic interference, leading to inaccurate measurement data. Therefore, based on the rotation characteristics of the moving contact during opening and closing, a method for measuring rotation angles in a straight line is proposed. A fiber optic angle sensor is designed according to the characteristics of reflective fiber optics to measure the rotation angle of disconnectors. The Mean Evolution Algorithm (MEA) is used to optimize the BP neural network model (MEA-BP) to compensate for the nonlinearity of the designed fiber optic angle sensor. Through experimental simulations, the nonlinearity compensation effect of the MEA-BP model is compared with that of the traditional BP neural network. The mean square error of nonlinearity compensation decreases from 0.0916 to 0.0211, and the fitting accuracy improves from 83.57% to 95.06%. The linear error decreases from 6.85% to 0.81%. An experimental platform using SBE27.5 kV/2000A disconnectors as the research object is established to test the accuracy of the fiber optic angle sensor. The experimental results show that the accuracy of the fiber optic angle sensor improves from 8.043% to 0.781% after nonlinearity compensation, indicating that this solution has higher accuracy and stronger feasibility.

## Introduction

High-voltage disconnect switches are essential electrical equipment in power grids. Their primary functions include voltage isolation and providing clear disconnection points during operation, ensuring the safety of power line maintenance personnel^1^. Therefore, monitoring the normal operation of high-voltage disconnect switches is crucial. Common detection methods for switch operation include motor current-based fault diagnosis, contact pressure detection, position detection using attitude sensors, auxiliary contact principles, image recognition, and optical sensing. Among these, Document^2^ proposes detecting mechanical faults through motor current variations using K-means algorithm to establish fault diagnosis models for determining whether operations are complete. However, since the completion status is reflected on the switch itself rather than the rotating shaft, this method has inherent limitations. Document^3^ suggests installing pressure sensors at contact finger springs to monitor pressure changes during operations. Document^4^ proposes using attitude sensors to measure positional angles during operations. Both methods employ electronic sensors, but challenges arise during installation: difficult power access and susceptibility to electromagnetic interference causing signal distortion. Document^5,6^ presents video image processing-based methods for identifying switch operation completion status. The method is highly sensitive to weather conditions, resulting in low recognition accuracy and susceptibility to electromagnetic interference during image transmission. As reported in reference^7^, an infrared temperature sensor is employed to detect the contact temperature of the isolating switch, thereby determining its open/closed state. While this approach can confirm the closed position of the isolating switch, it fails to identify the open state.

In summary, existing isolation switch status monitoring methods each have their limitations. Specifically, when focusing on direct rotation angle measurement, traditional high-precision angle sensors (such as photoelectric encoders and potentiometers) face fundamental challenges in high-voltage isolation switch applications. While photoelectric encoders offer high accuracy, their internal electronic components and signal transmission are highly susceptible to interference from strong electromagnetic fields, leading to signal distortion or damage. Potentiometers (variable resistors) use contact-based measurements, which suffer from mechanical wear, limited lifespan, and risks of insulation failure and electrical arcing in high-voltage environments. Additionally, magnetic measurement devices like Hall sensors are inherently sensitive to magnetic fields, making it difficult to ensure reliable performance in complex electromagnetic environments. The common challenge for these methods lies in achieving long-term stable, reliable, and high-precision direct angle measurement under harsh conditions with high voltage and strong electromagnetic interference. This contradiction between “measurement reliability” and “environmental harshness” represents a significant gap in current technology.

Based on this, the paper adopts an optical fiber sensor with good insulation properties, strong anti-electromagnetic interference capability, and corrosion resistance to design an optical fiber angle sensor suitable for detecting the rotation angle of the moving contact of a high-voltage isolator. By using optical fiber ranging methods to convert the rotation angle into linear distance, the relationship between the rotation angle of the isolator and the output voltage of the sensor is obtained, thereby calculating the rotation angle of the blade. To address the defects in the sensor design and the nonlinear issues caused by the environment, a thought evolution algorithm is used to optimize the BP neural network model for nonlinear compensation. The designed optical fiber angle sensor is installed at the rotation end of the moving contact of the SBE27.5 kV/2000A isolator for testing, and the measurement results are compared with those of existing high-precision angle sensors to determine whether the designed optical fiber angle sensor meets the requirements.

To clearly distinguish this study from existing research, the main contributions of this paper can be summarized as follows:

Innovation in measurement principle: A reflective fiber-optic displacement sensing structure combined with a rack-and-pinion linear–angular conversion mechanism is proposed. The angular displacement is converted into linear displacement, allowing the sensing unit to be located away from high-voltage components, thereby improving electrical isolation and electromagnetic immunity.

Innovation in structural integration design: The mechanical transmission radius (*r* = 15.5 mm) and reflective mirror configuration are optimized to enlarge the effective linear measurement range while reducing mechanical interference and alignment sensitivity.

Innovation in nonlinear compensation algorithm: The Mind Evolutionary Algorithm (MEA) is introduced to globally optimize the weights and thresholds of a BP neural network. Compared with traditional BP and polynomial fitting methods, the proposed MEA-BP significantly reduces nonlinear error and improves linearity and fitting accuracy.

## Measurement scheme of rotary angle of isolating switch

(1) Analysis of the rotation angle characteristics.

**Fig. 1:**
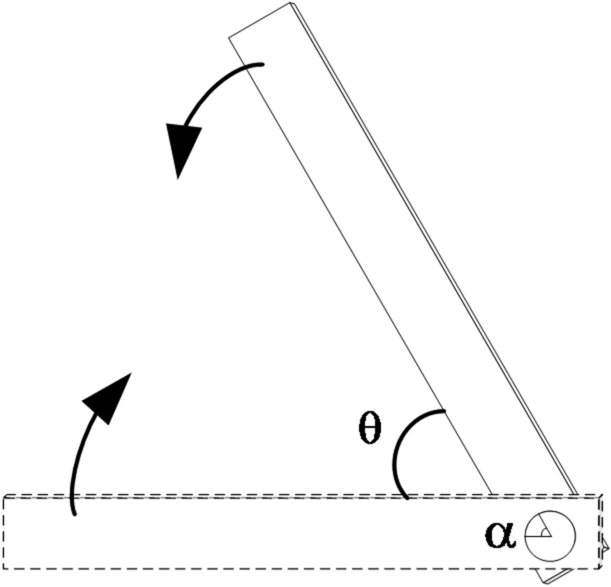
Schematic diagram of opening and closing isolation switch.

As shown in Fig. 1, During opening and closing operations, the moving contact is rigidly connected to and rotates coaxially with the pivot shaft. Therefore, a strict one-to-one correspondence exists between the mechanical rotation of the shaft and the operational angle of the contacts. This fundamental relationship is expressed as:

1 $$\alpha =\theta$$

where, *α* denotes the mechanical rotation angle of the pivot shaft, *θ* represents the electrical opening/closing angle between the moving and stationary contacts. On this basis, the paper transfers the measuring point from the high-voltage knife switch to the position convenient for installation and maintenance, so as to meet the requirements of electrical insulation and anti-interference in high-voltage environment.Thus, by measuring *α*, the operational state *θ* of the isolator can be accurately determined.

(II) Angle Measurement Design.

High-voltage disconnect switches operate in complex environments with intense electromagnetic radiation, which can cause electromagnetic interference (EMI) to surrounding electronic devices^8^. Traditional sensors for measuring switch operations are predominantly electronic types, yet they suffer from issues like poor insulation and weak EMI resistance. Direct installation of these sensors on disconnect switches also poses challenges in power supply. To address these issues, this paper proposes a fiber-optic angle sensor with superior insulation, strong EMI resistance, and corrosion resistance, specifically designed for detecting the rotational angle of high-voltage disconnect switch moving contacts. By adopting the “straight-line substitution” principle, the sensor converts the moving contact’s rotation angle into measurable linear displacement, effectively transforming angular measurements into linear distances through fiber-optic ranging technology.

**Fig. 2:**
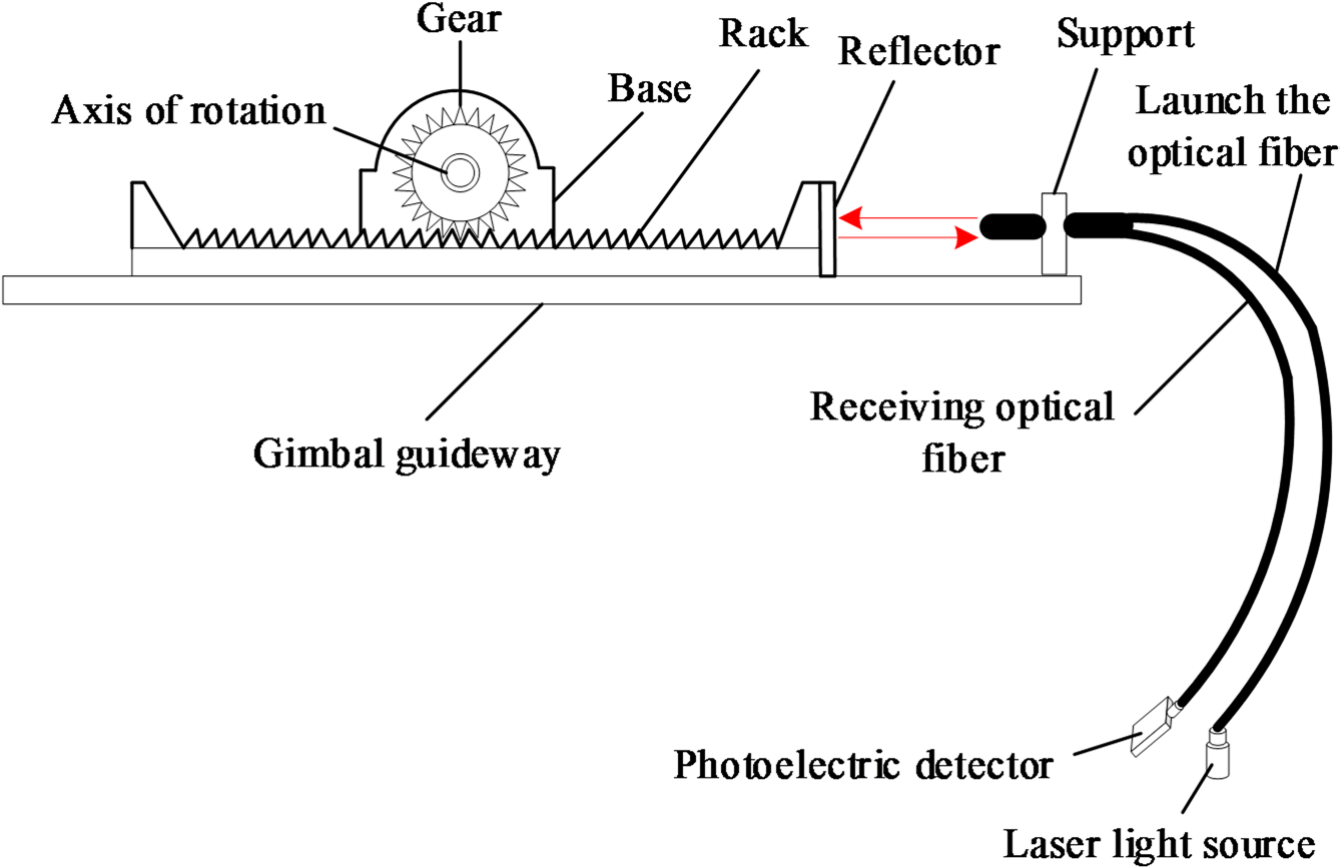
Schematic diagram of optical fiber Angle sensor.

As shown in Fig. 2, the fiber angle sensor designed in this paper is based on the principle of reflective intensity modulation. Its mechanical structure includes a base, sliding rails, a gear coaxially fixed with the isolation switch’s rotating shaft (radius *r* = 15.5 mm), a rack meshed with the gear, and a high-reflection mirror (reflectivity *R* ≈ 0.95) mounted on the rack. A commercial fiber displacement sensor probe (model: IFS-M30M-2, Keyence Corp) is fixed via a bracket, with its end face vertically aligned with the mirror. The sensor integrates a laser diode (wavelength 650 nm), transmitting and receiving fibers (62.5/125µm multimode fiber), and a photodetector. When the moving contact rotates, the shaft drives the gear, which in turn moves the rack and mirror linearly along the rails, thereby changing the distance *L* between the mirror and the probe’s end face. Light from the laser is transmitted through the transmitting fiber to the mirror, and the reflected light is collected by the receiving fiber. The photodetector converts the received light intensity into the corresponding output voltage u.

For the reflective dual-fiber probe used, the relationship between the received optical power Pr and the distance *L* can be described by the following empirical model:

2 $${P_{\mathrm{r}}}(L)=\frac{C}{{{L^2}+K}} \cdot R \div \eta (T,H) \cdot \xi (A)$$

Here, *C* is a constant related to the light source power and fiber parameters, *K* is a constant associated with the probe’s optical design, and *η*(*T*, *H*) and *ξ*(A) are the influence factors of ambient temperature *T*, humidity *H*, and stray light *A*, respectively. The output of the photoelectric conversion process is voltage:

3 $$u=G \cdot {P_{\mathrm{r}}}+{u_{0f}}$$

Here, *G* denotes the overall photoelectric gain, while ${u_{0f}}$ encompasses dark current and circuit drift.

The displacement *L* is linked to the rotational angle θ (in radians) of the rotating shaft via a gear-rack mechanism, ideally expressed as *L* = *r·θ*. When accounting for mechanical defects (e.g., gear clearance or rail friction), the actual relationship incorporates an error term *e*_m_(θ).

The complete characteristic equation for the sensor input (angle *θ*) and output (voltage *u*) can be obtained.

4 $$u(\theta )=G \cdot \left[ {\frac{C}{{{{\left( {r \cdot \theta +{e_m}(\theta )} \right)}^2}+K}} \cdot R \cdot \eta \cdot \xi } \right]+{u_{0f}}$$

**Fig. 3:**
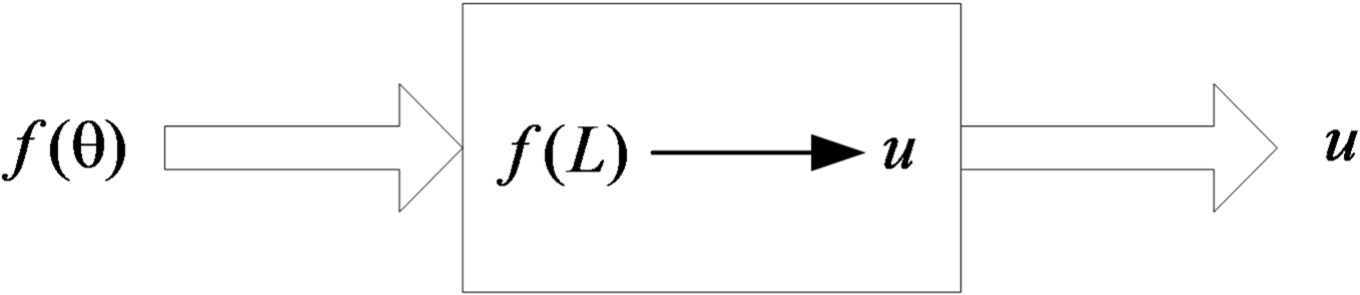
The relationship between *θ* and *u*.

By measuring the output voltage *u* of the photosensitive demodulation corresponding to each displacement *L*, and treating the relationship between *L* and u as a black box, one can directly measure the angle *θ* to obtain the relationship between *θ* and *u*. The output voltage *u* is shown in Fig. 3.

**Fig. 4:**
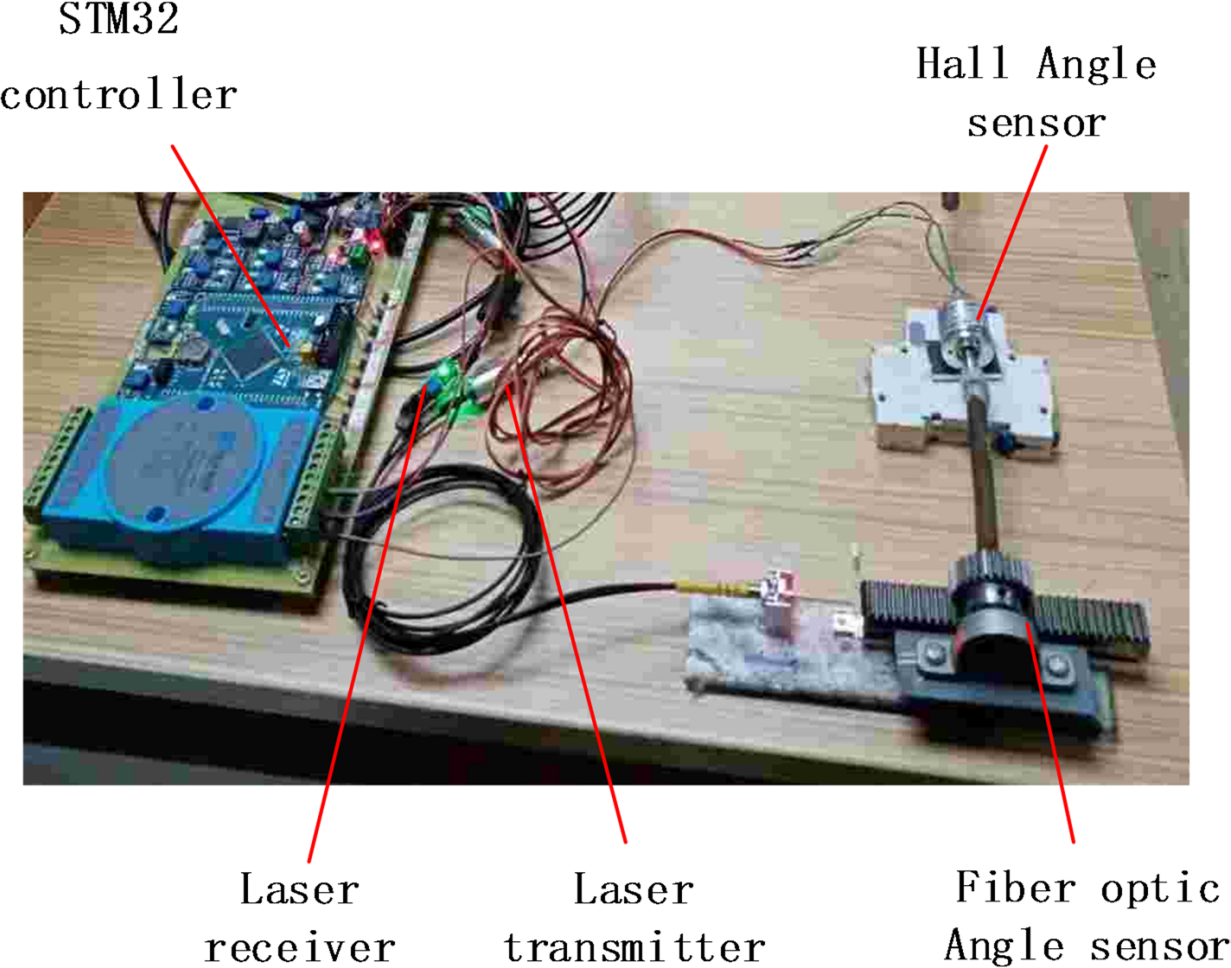
Experimental Platform.

A data acquisition platform for the designed fiber optic angle sensor was set up as shown in Fig. 4. The core sensing component is a commercial reflective intensity-modulated fiber optic displacement sensor (Model: IFS-M30M-2, Keyence Corp., Japan). Its probe contains a pair of transmitting and receiving fibers. The built-in amplifier outputs an analog voltage signal (0–5 V) corresponding to the distance between the probe tip and the reflective target. The sensor has a specified measurement range of 2–30 mm, a repeatability of ≤ 1.0 μm, and operates at a red laser wavelength of 650 nm. Prior to assembly into the angle sensor mechanism, the fiber optic displacement sensor probe was independently calibrated. Using a high-precision translation stage (PI, M-110.1DG) to control displacement and a data acquisition card (NI, USB-6361) to record output voltage, the static characteristic curve u-L (Voltage-distance) was obtained and verified to be consistent with the manufacturer’s specifications within the working range. When the moving contact of the isolating switch rotates from 0° to 90°, the corresponding total linear displacement of the reflector relative to the fiber optic probe is 24.3 mm.

**Fig. 5:**
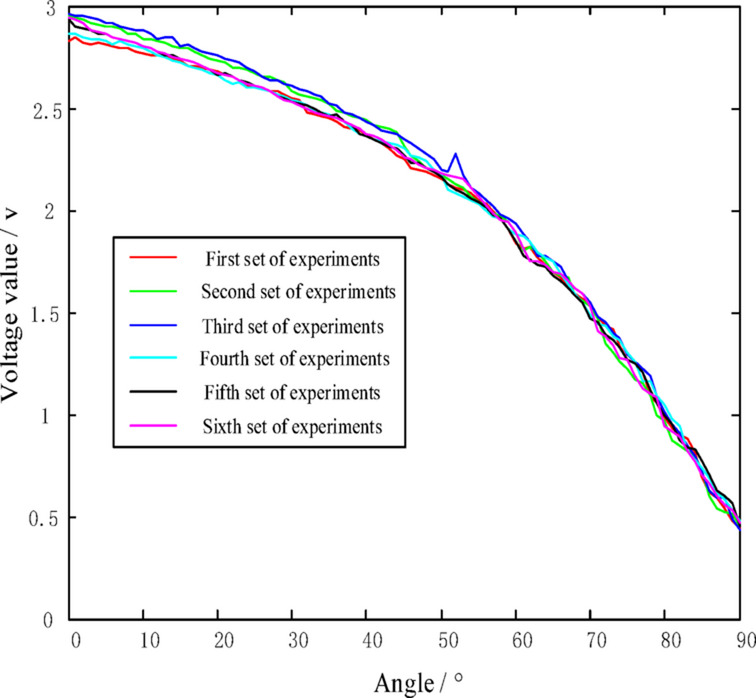
The characteristic curve between *θ* and *u*.

The test data include the output voltage measured at each angle within the 0°~90° range. After the output voltage stabilized at each angle point, data sampling was performed at 100 Hz with six round-trip measurements. The experiment was conducted in a constant temperature and humidity chamber under the conditions of 25 ± 2 °C and 50 ± 10% relative humidity. The characteristic curve of θ versus u was plotted, as shown in Fig. 5.

As can be seen from Fig. 5, the characteristic curves between θ and u obtained from the six experiments do not exhibit ideal linearity. The nonlinear behavior observed in the experimental u-θcharacteristic curve stems from three primary sources: (1) the inherent nonlinear response dictated by optical principles (specifically the (*L*2 + *K*)^−1^; (2) mechanical nonlinearity introduced by *e*_m_(θ); and (3) environmental drift and interference contained in *η*(*T*,*H*), *ξ*(*A*), and *u*_0f_. Consequently, the target to be compensated constitutes a complex, coupled nonlinear function, which provides a theoretical foundation for subsequent applications of powerful nonlinear mapping tools such as MEA-BP neural networks.

## Simulation of nonlinear compensation model

Nonlinear compensation for sensors can be implemented through hardware circuits or software approaches. However, hardware solutions are susceptible to electromagnetic interference, which increases product costs. This paper adopts a software-based approach to perform nonlinear compensation for the fiber optic angle sensor in this design. Among commonly used nonlinear compensation algorithms, the Backpropagation (BP) neural network demonstrates strong nonlinear mapping capabilities, making it suitable for sensor compensation. However, BP neural networks exhibit slow convergence speeds, are prone to local minima, and may yield local optima. Additionally, initial parameters such as connection weights between network layers and thresholds that determine fitting quality are predetermined during modeling, lacking selection criteria^9–11^, thus requiring optimization. The MindEvolutionaryAlgorithm (MEA) demonstrates exceptional global optimization capabilities. Therefore, this paper proposes a Mind Evolutionary Algorithm (MEA-BP) tailored to the operational characteristics of fiber optic angle sensors, aiming to enhance both convergence speed and accuracy of the BP neural network. In this design, the Mind Evolutionary Algorithm primarily focuses on optimizing connection weights and thresholds within the BP neural network framework.

(1) Constructing BP Neural Network Model.

Let *n* = *f*(*u*) be the characteristic function of the fiber optic angle sensor. Due to processing defects of its own mechanical components, as well as temperature and humidity effects, *f*(*u*) becomes a nonlinear function. Therefore, a compensation model function *P* = *f*(*n*) is applied after the output n to compensate for *f*(*u*). For BP neural networks, increasing the number of hidden layers will lead to longer network training times and lower learning efficiency^12^. Previous simulation experiments have shown that when the number of hidden layer nodes can be freely set, a three-layer feedforward neural network can approximate any nonlinear function, and any precision value corresponds to an output angle value. Therefore, the BP neural network model structure used in this paper consists of one input layer and one output layer. The number of hidden layers needs to be determined by considering the network training time. In this case, less training time is better, so the number of neurons in the hidden layer can be obtained through simulation with training samples. Moreover, the activation function and learning function are important components of each layer in the BP neural network model^13^. The output layer uses the bipolar S function (tansig) and the linear transfer function (purelin) for learning. The function chooses the weight/threshold learning function (learnngdm). The activation function is used to introduce nonlinear factors to improve the neural network model’s expressive capability.

(2) Determination of Network Structure.

The Tansig function operates within the [-1,1] threshold range, requiring data preprocessing through normalization to map values to this interval. Based on the rotational characteristics of the isolating switch’s moving contact during operation, the optical fiber angle sensor collects voltage readings at 1° intervals across the 0 ~ 90° range, with 12 experiments conducted. This yielded 1080 sample data sets. Table 1 presents selected angular voltage measurements.

**Table 1:** Part of training sample data.

Angle θ/°	0	1	2	3	4	5
Voltage value u /v	2.83	2.85	2.82	2.81	2.82	2.81
……	……	……	……	……	……	……
Angle *θ* /°	85	86	87	88	89	90
Voltage value u /v	0.7	0.62	0.59	0.54	0.48	0.44

From the collected data, 1000 groups were randomly selected for normalization. Among the processed data, 450 groups were randomly chosen as training samples and 50 as test samples. Using MATLAB, the network was trained and predicted on these datasets to determine error precision and iteration counts for different hidden layer neuron counts. As shown in Table 2, the network achieved the lowest iteration count and highest error precision (0.0160) when the hidden layer neuron count was 7. Therefore, the hidden layer neuron count was set to 7.

**Table 2:** Error precision and iteration times of different number of neurons in hidden layer.

Number of neurons	Mean square error	Number of iterations	Number of neurons	Mean square error	Number of iterations
3	0.0876	27	8	0.1017	12
4	0.1818	62	9	0.1252	32
5	0.1819	10	10	0.1536	12
6	0.1618	11	11	0.1536	14
7	0.0160	7	12	0.2458	12

**Fig. 6:**
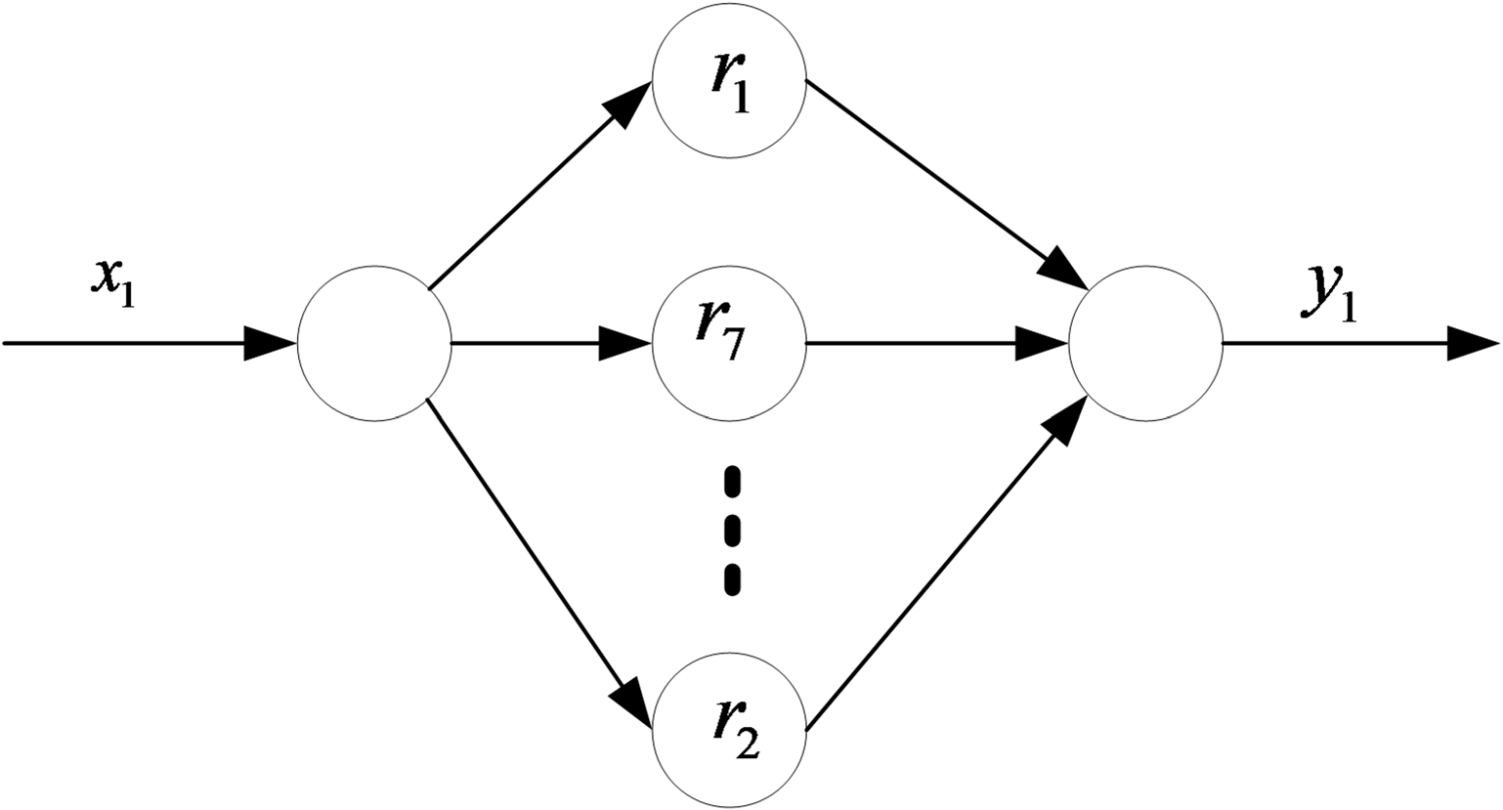
Structure diagram of the BP neural network. BP neural network structure diagram.

In conclusion, the BP neural network in this paper has one input layer, seven hidden layers and one output layer. The BP neural network structure for nonlinear compensation is shown in Fig. 6.

(3) Nonlinear Compensation Model of MEA Optimized BP Neural Network.

The MEA algorithm’s advantages can compensate for the limitations of BP neural networks. This study employs MEA to optimize the connection weights and thresholds of BP neural networks. Specifically, based on the obtained BP neural network architecture, the MEA solution space is mapped to the encoding space. Each code corresponds to a solution (individual), and the reciprocal of the mean square error (MSE) of training samples is used as the scoring function for both individuals and the population. MEA iteratively outputs optimal individuals, which are then used as initial weights and thresholds to train the BP neural network^14,15^. The specific optimization process is as follows:

1) The collected sample data undergoes preprocessing to construct a BP neural network architecture, mapping the MEA solution space into a coding space. With 1 input layer neuron, 7 hidden layer neurons, and 1 output layer neuron, the resulting encoding length is:

5 $$s=1 \times 7+7 \times 1+7+1=22$$

2) Population generation. Randomly generate N groups as the initial structure population, with each group containing N elements representing individual BP neural network $0,1, \ldots ,m$architectures. Each element (individual) comprises multiple nodes from the hidden layer, thereby $j - 1=0,1, \ldots ,7$randomly forming a structure $({p_{11}},{p_{12}}, \ldots ,{p_{18}})$population^16^. In the obtained BP neural network architectures, let denote the number of nodes in the hidden layer, and the number of neurons in the hidden layer be, where represents the number of neurons in the hidden layer. The individual score can then be expressed as:

6 $$P=\left[ {\begin{array}{*{20}{c}} {{p_{11}}}&{{p_{12}}}& \cdots &{{p_{18}}} \end{array}} \right]$$

In the context of neural network encoding, the parameter matrix P records the information of each individual (network structure), where each parameter position is randomly generated.

3)Population Optimization and Objective Function. The MEA algorithm randomly generates X groups of individuals (i.e., X distinct combinations of network weights and thresholds) within the solution space. Its core optimization objective is to find a set of weights and thresholds for the BP network that minimizes error on the training dataset. Each individual (a weight-threshold combination) is then fed into the BP network’s forward propagation process to evaluate its performance across all training samples. In this study, mean squared error (MSE) serves as the objective function to measure individual performance. Lower MSE values indicate more accurate predictions from the network constructed with these parameters, reflecting superior individual performance. To align with MEA’s evaluation principle of “higher scores mean better results,” the fitness score of an individual is defined as the reciprocal of MSE. Specifically, for an individual, the MSE of its corresponding BP network on the training set is:

7 $$MSE=\frac{1}{N}\sum\limits_{{i=1}}^{N} {{{({t_i} - {y_i})}^2}}$$

Here, ti denotes the ideal angle output of the i-th sample, yi represents the network’s actual angle output, and N is the total number of training samples. The fitness score F for this individual is defined as:

8 $$F=\frac{1}{{MSE+\varepsilon }}$$

A minimum constant $\varepsilon$is introduced here to prevent division by zero errors. The MEA algorithm ranks all individuals based on their fitness score F, with the highest-scoring individual designated as the temporary winner. Through iterative execution of subsequent convergence and divergence operations, the MEA algorithm continuously searches for individuals with higher fitness scores (i.e., lower MSE), thereby progressively approaching the optimal network parameters.

4) Similarity operation of subpopulations. The similarity operation generates individuals centered around high scores and produces individuals in the form of a normal distribution, forming subgroups M + T^17^. Any individual in a subpopulation can be represented as N(µ, Σ), where µ is the center vector of the normal distribution and Σ is the covariance matrix of the normal distribution. Therefore, the BP neural network weight parameters with the highest score serve as the center vector µ.

5) Divergence operation of subpopulations^18^. That is, the global winner is selected from i winning subgroups. In this paper, the simplex optimization method is used for discretization operations. Let the first *i* + 1 winning individuals be denoted as *Q*_*1*_, *Q*_*2*_, *.*,* Q*_*i*_, *Q*_*i+1*_, then the function value of the (*i* + 1)th vertex is calculated to obtain the worst point QW and the centroid Qm, where:

9 $${Q_m}=\frac{{{Q_1}+{Q_2}+ \cdots +{Q_i}+{Q_{i+1}} - {Q_w}}}{i}$$

Then calculate the reflection point *Q*_*r*_ and *Q*_*w*_:

10 $${Q_r}={Q_m}+({Q_m} - {Q_w})\;{Q_r}={Q_m}+({Q_m} - {Q_w})$$

The new simplex obtained through the reflection point *Q*_*r*_ is guaranteed to have at least one vertex that is better than a vertex of the initial simplex. The process is repeated until convergence is achieved.

**Fig. 7:**
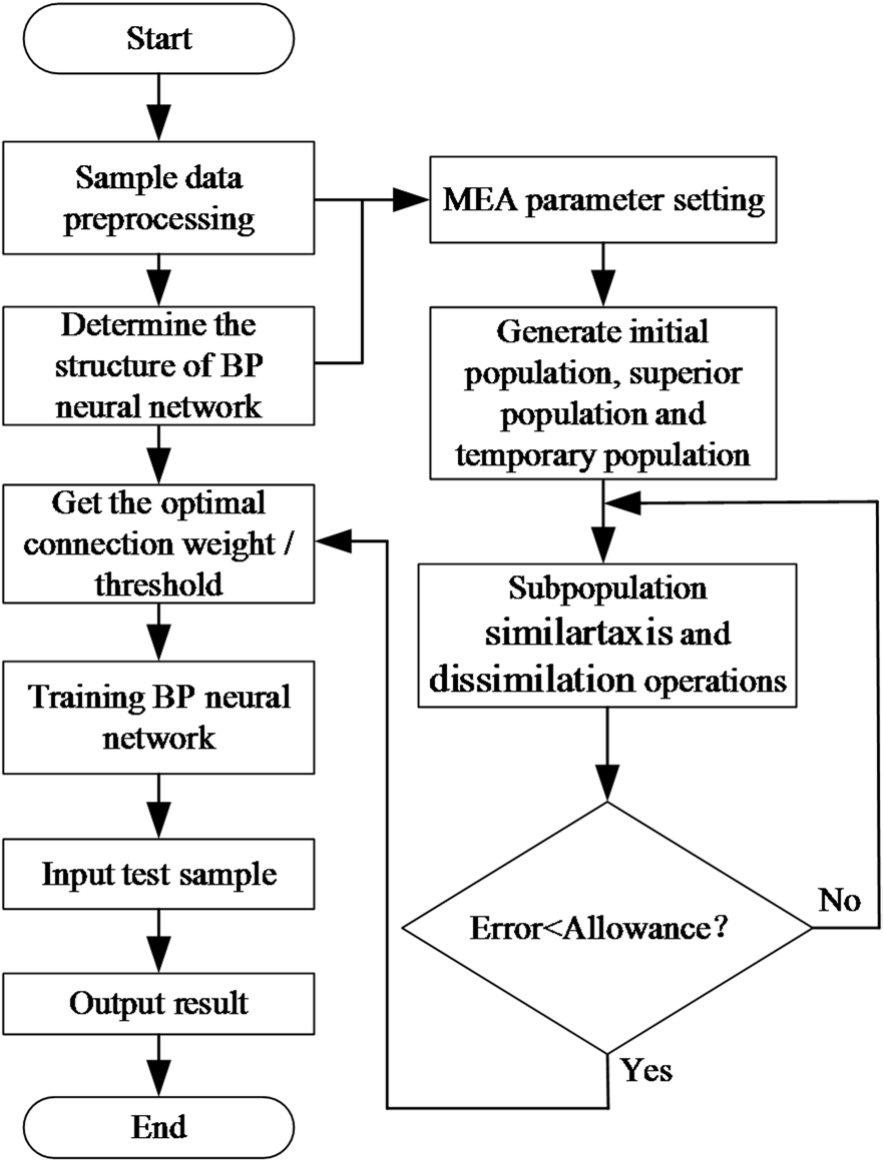
Flowchart of MEA-BP model algorithm.

6) Determine whether the convergence criteria are met. If the convergence criteria are not met, continue with the homogenization and heterogenization operations. Once the convergence criteria are satisfied, MEA ends the optimization process and identifies the optimal individual according to the encoding rules, which corresponds to the weights and thresholds of the BP neural network. The BP neural network then uses the optimized weights and thresholds for training and learning, ultimately producing the desired results. The flowchart of the MEA-BP model algorithm is shown in Fig. 7.

(IV) Results of Nonlinear Compensation.

To verify the necessity of compensating the nonlinear problem of the fiber angle sensor with MEA-BP model, the remaining 500 sets of data (450 sets of training set and 50 sets of test set) were simulated and analyzed by BP neural network model and MEA-BP model respectively, and the corresponding compensation models were obtained. The output accuracy and fitting degree of the two models after compensation for the fiber angle sensor were analyzed and compared.

1. BP neural network compensation.

The performance function of a BP neural network quantifies its output accuracy and determines the error magnitude. Commonly used performance metrics include Mean Square Error (MSE), Mean Absolute Error (MAE), and Mean Absolute Percentage Error (MAPE). Among these, MSE effectively measures the network’s approximation to the target function^19,20^, which is why this study adopts MSE as the performance metric to evaluate the network’s output precision.

**Fig. 8:**
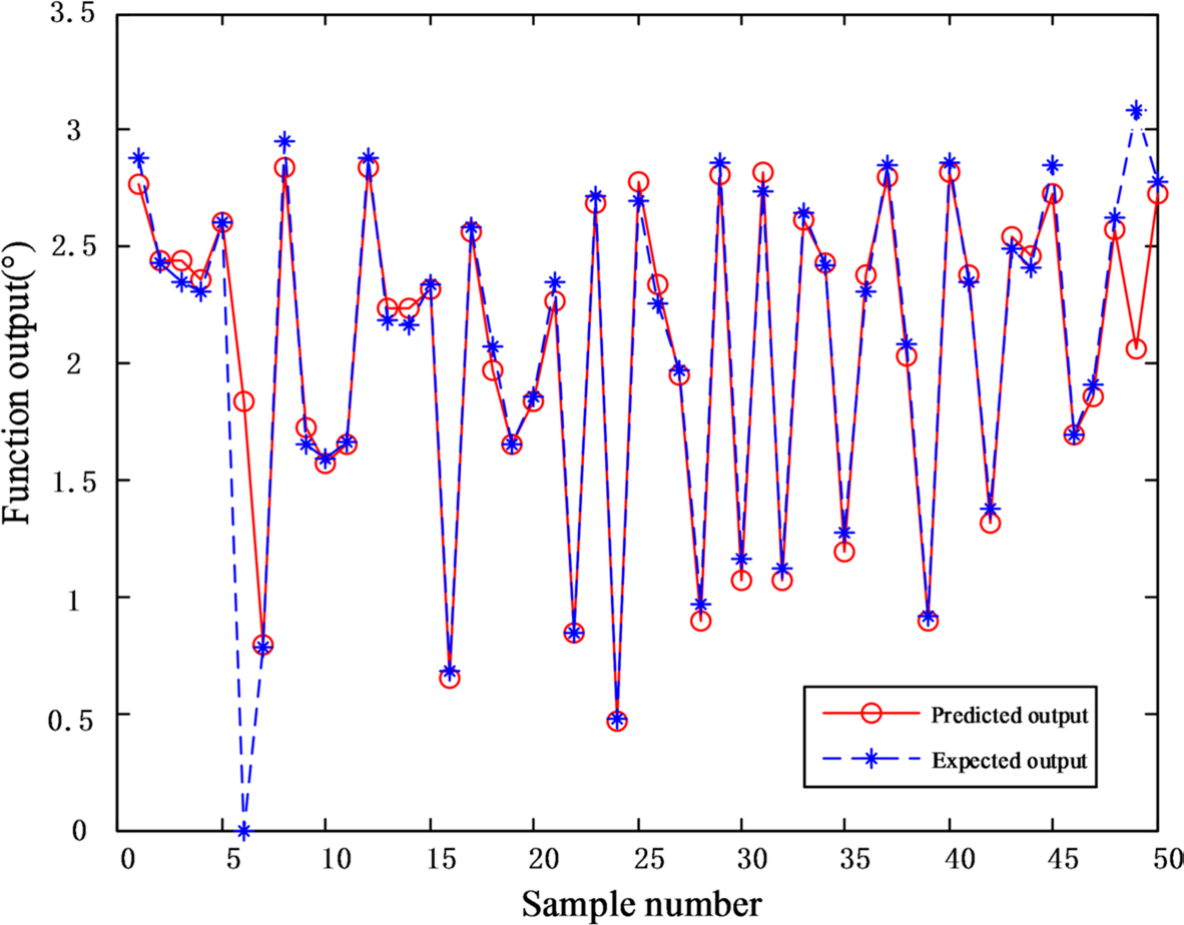
Prediction error of BP neural network.

**Fig. 9:**
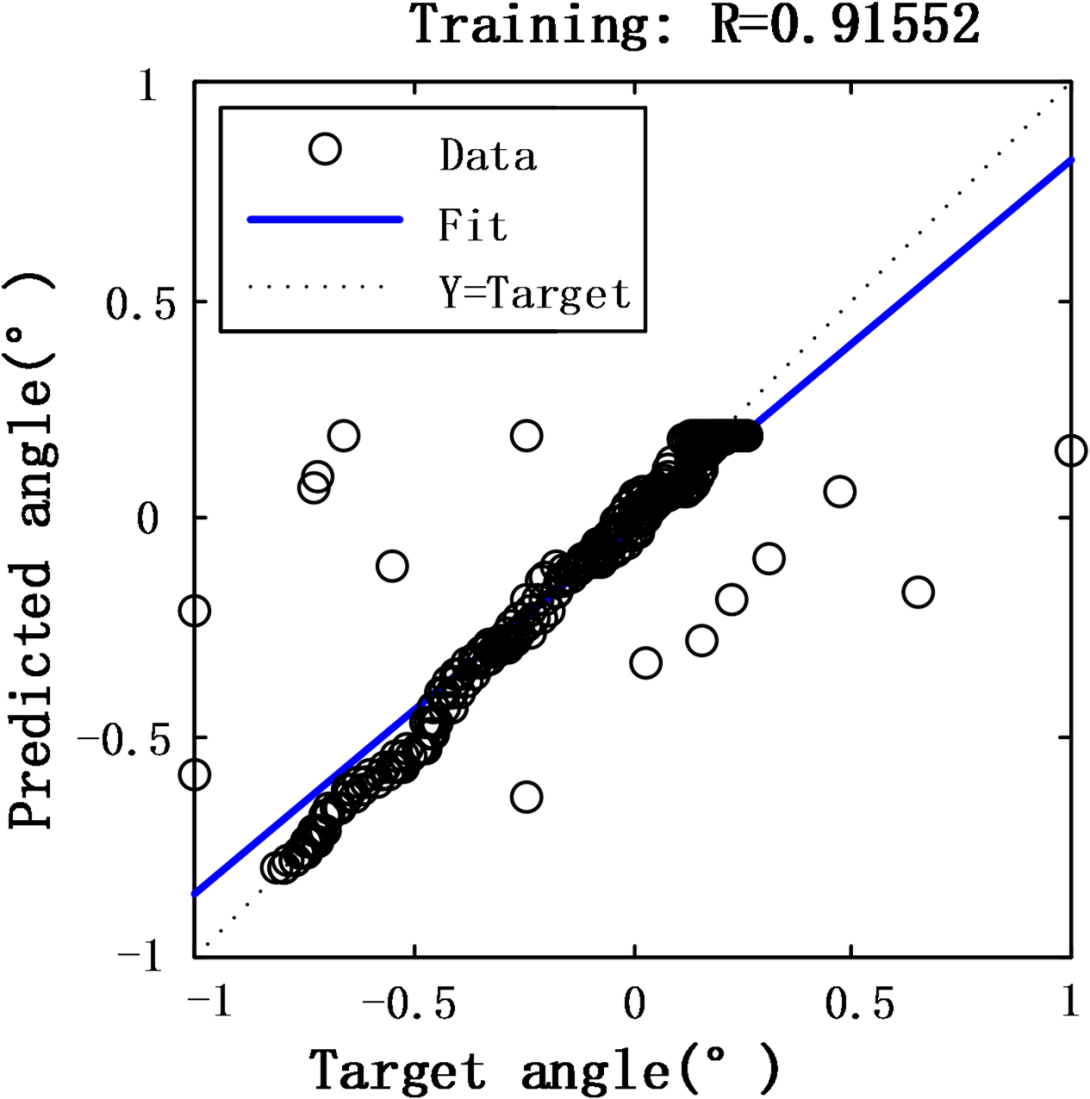
BP neural network fit degree.

As shown in Figs. 8 and 9, the BP neural network achieves a root mean square error of 0.0916 and an 83.57% fitting accuracy when performing nonlinear compensation for the fiber angle sensor.

**Fig. 10:**
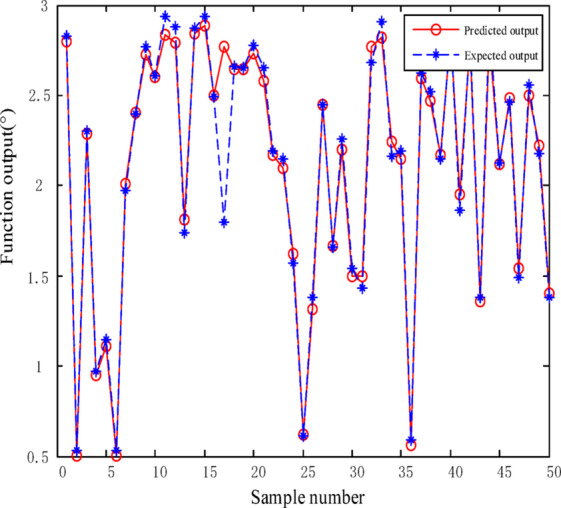
Prediction error of MEA-BP model.

**Fig. 11:**
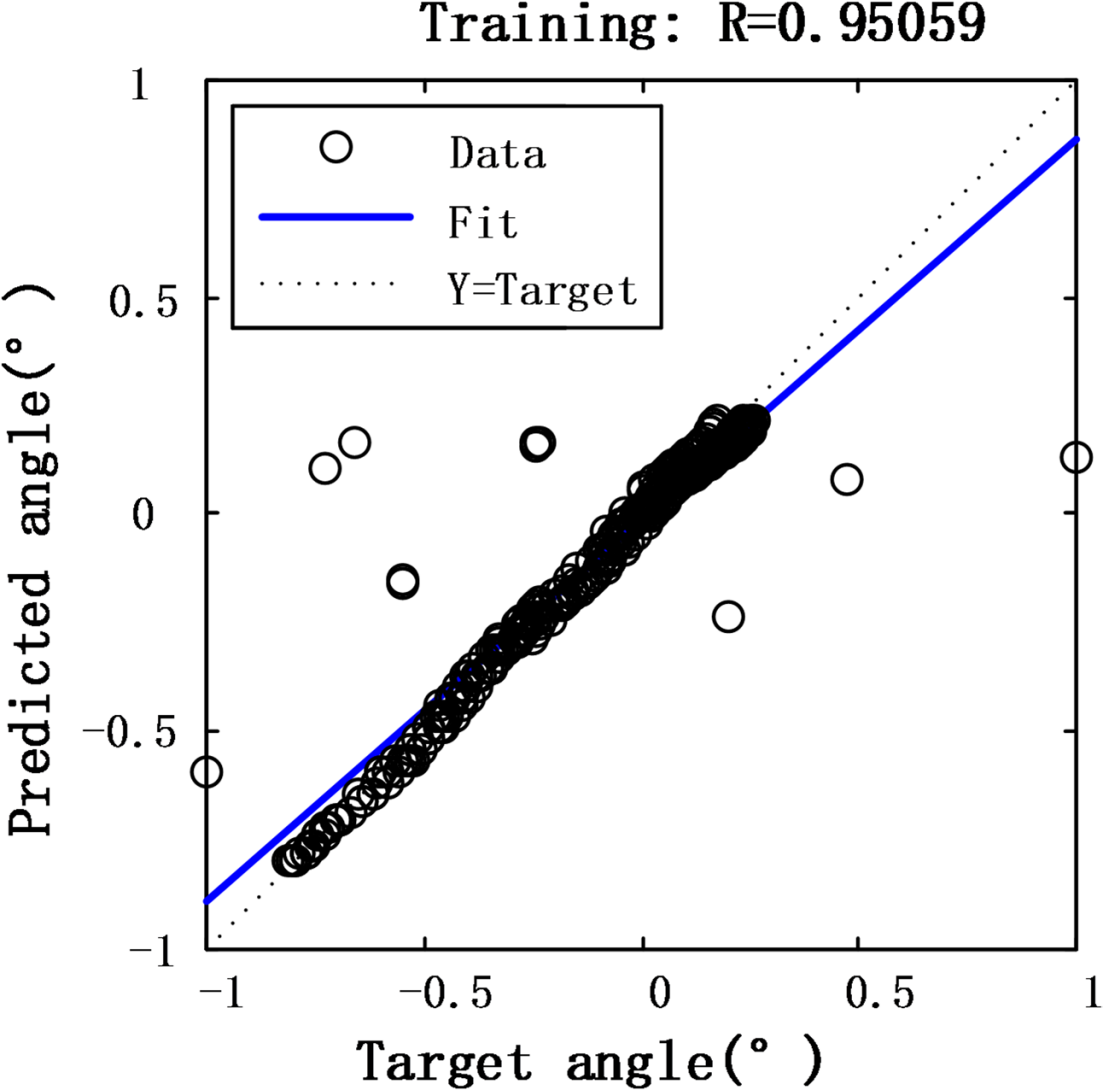
The fitting degree of MEA-BP model.

Figures 10 and 11 demonstrate the performance of a BP neural network model optimized using the MEA algorithm to compensate for nonlinearities in the designed fiber angle sensor. The results show that after optimizing the network’s weights and thresholds with the MEA algorithm, the mean square error was reduced to 0.0211, achieving a fitting accuracy of 95.06%.

Compared with the WOA-BP method in reference^21^, which optimizes BP neural networks via the Whale Optimization Algorithm for global search and yields a maximum error of 0.38%, our proposed MEA-BP approach delivers better compensation performance in this nonlinear sensing scenario with a much lower maximum error of 0.0211 under the same evaluation criteria; this is because WOA-BP only focuses on population-wide stochastic exploration, while the MEA introduces subgroup evolution and similartaxis mechanisms to balance global exploration and local refinement, making MEA-BP more adaptive to the complex nonlinear characteristics of fiber-optic angle sensors caused by mechanical transmission deviations, environmental humidity changes and external light source interference. In addition, unlike the nonlinear correction method for microwave power sensors in reference^22^, which relies on hardware calibration and system optimization with a 3.5% maximum compensation error and suffers from high system complexity, increased costs and limited adaptability to structural or environmental changes, our MEA-BP is a purely software-based nonlinear compensation solution that avoids extra hardware modification costs, boasts stronger flexibility for different nonlinear conditions, and achieves significant error reduction and improved stability while keeping the system simple.

Eight data points in Fig. 11 deviate from the linear fitting line. This deviation may be attributed to mechanical backlash in the rack-and-pinion structure, slight reflective mirror misalignment, or transient environmental disturbances.

2. Linear analysis.

The linear degree of the traditional BP neural network and the MEA-BP compensation model^23^ are compared by using the linear formula.

11 $${\delta _L}=\frac{{|\Delta {L_m}|}}{{Y(FS)}} \times 100\%$$

In the formula, |Δ*L*_*m*_| is the maximum deviation between the output of the fiber optic angle sensor and the measured value of the calibration curve, and Y(FS) is the maximum value at full-scale output. Using the designed fiber optic angle sensor to collect experimental data for 10 angle calibration values (0°, 10°, … 90°), the collected data were used as the test set and input into the BP network model and the MEA-BP network model to calculate the predicted results, as shown in Table 3.

**Table 3:** Predictive outputs of BP network model and MEA-BP model.

Angle calibration value /°	0	10	20	30	40
$U/{\mathrm{v}}$	2.93	2.80	2.66	2.53	2.36
${U_x}/{\mathrm{v}}$	2.95	2.79	2.67	2.53	2.39
${\Delta _1}$	-0.02	0.01	-0.01	0.00	-0.03
${U_y}$	2.92	2.78	2.66	2.54	2.38
${\Delta _2}$	0.01	0.02	0.00	-0.01	-0.02
Angle calibration value /°	50	60	70	80	90
$U/{\mathrm{v}}$	2.16	1.85	1.47	1.00	0.45
${U_x}/{\mathrm{v}}$	2.12	1.93	1.55	0.94	0.28
${\Delta _1}$	0.04	-0.08	-0.08	0.06	0.17
${U_y}$	2.16	1.87	1.49	0.99	0.46
${\Delta _2}$	0.00	-0.02	-0.02	0.01	-0.01

Note: *U* is the actual measured value, *U*_*x*_ and Δ_1_ are the predicted output value and deviation of the BP network model, and U_y_ and Δ_2_ are the predicted output value and deviation of the MEA-BP network model, respectively.

As can be seen from Table 3, the maximum deviation of the BP network model occurs when the input angle is 90°. At this time, ${\Delta _1}=|{U_x} - U|=0.17v$, the difference between the measured voltage at a full-scale output angle of 90° and the measured voltage at 0°, represents the linearity of the BP network model as follows:

12 $${\partial _{L1}}=\frac{{0.17}}{{2.48}} \times 100\% =6.85\%$$

The maximum deviation of the MEA-BP network model occurs when the input angles are 10°, 40°, 60°, and 70°, at which point ${\Delta _2}=|{U_x} - U|=0.02v$. The linearity of the MEA-BP network model is:

13 $${\partial _{L2}}=\frac{{0.02}}{{2.48}} \times 100\% =0.81\%$$

Comparing Eqs. (11) and (12), the nonlinear degree of the fiber angle sensor after nonlinear compensation is improved from 6.85% to 0.81%, which is close to the nonlinear degree of the existing high-precision Hall angle sensor (0.3%)^24^. This indicates that the linearity of the fiber angle sensor can be significantly improved after using the MEA-BP model for compensation.

(V) Performance Comparison of Compensation Methods.

To objectively evaluate the effectiveness of the MEA-BP model and demonstrate its advantages over traditional simple methods, this section compares the compensation results of the aforementioned MEA-BP model with those of other typical compensation methods applied to the same dataset. The comparison methods include: quadratic polynomial fitting, cubic spline interpolation lookup table, and traditional BP neural network. The fitting accuracy and linear error comparison results of each method are shown in Table 4.

**Table 4:** Comparison of Performance of Different Nonlinear Compensation Methods.

compensating process	Brief principle	fitting accuracy	linear error	Key indicator source/definition
quadratic polynomial fitting	The least square quadratic fitting is carried out to the u-θ data points.	90.2%	7.4%	Fitting based on the same training set and calculating on the test set. Insufficient for complex nonlinear approximation.
Look up table (linear interpolation)	Store lookup tables with intervals of 1, and perform linear interpolation for intermediate values.	88.5%	5.3%	The interpolation point density is 91. The inherent nonlinearity of the data cannot be overcome, resulting in significant interpolation errors.
Traditional BP Neural Network	The standard BP algorithm (with the same structure as Sect. 2.2).	83.57%	6.85%	As shown in the results of Eq. (12), the algorithm is prone to local optima, resulting in performance limitations.
MEA-BP neural network	The initial weights and threshold of BP were optimized by MEA.	95.06%	0.81%	As shown in the results of Eq. (13), global optimization significantly improves performance.

To further validate the superiority of the proposed MEA-BP model, additional comparisons were conducted with two widely used evolutionary optimization algorithms: Genetic Algorithm optimized BP (GA-BP) and Particle Swarm Optimization optimized BP (PSO-BP).For fairness, all optimization algorithms were implemented using the same BP network structure (1–7–1), identical training and test datasets, and the same stopping criteria. The population size and maximum iteration number were set consistently to ensure comparable computational conditions.The comparison results are summarized in Table 5.

**Table 5:** Comparison of evolutionary optimization algorithms for BP weight initialization.

Algorithm	Fitting Accuracy	Linear Error	Average Iterations to Convergence	Stability (MSE Variance)	Key Characteristics
GA-BP	92.8%	2.4%	120	Moderate	Strong global search but slow convergence
PSO-BP	93.6%	1.8%	85	Good	Fast convergence but prone to premature convergence
MEA-BP	95.06%	0.81%	70	Excellent	Balanced global exploration and local refinement

As shown in Table 5,These results confirm that although GA and PSO are effective evolutionary optimization algorithms, MEA provides a better balance between exploration and exploitation for the nonlinear compensation problem addressed in this study.

## Analysis of experimental results

**Fig. 12:**
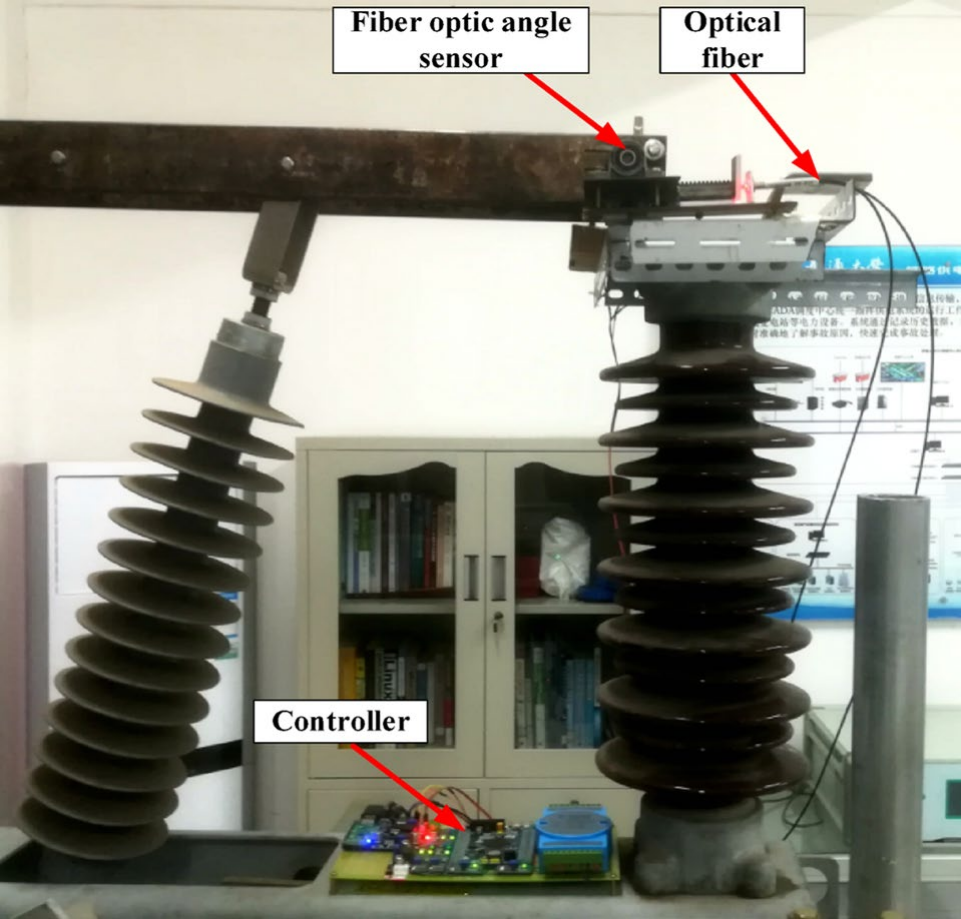
System installation inspection diagram. system installation check diagram.

To validate the reliability of the fiber-optic angle sensor designed in this study, comparative tests were conducted on an SBE27.5 kV/2000A open switch. The designed sensor was mounted on the rotating end, with a high-precision Hall-effect angle sensor (model: AS5048A, Austria’s AMS AG) used as a reference. This Hall sensor features a full 360-degree range, 14-bit resolution (0.022°), and a typical nonlinear error of ± 0.3% (as specified in the data manual). The sensor was directly coupled to the rotating shaft to obtain the reference angle value θ. Both sensor outputs were fed into the controller. The experiments were performed under laboratory conditions with an ambient temperature of 25 ± 2 °C and relative humidity of 50 ± 10%. The MEA-BP model algorithm was programmed into the controller to enable software-based nonlinear compensation. Figure 12 illustrates the system installation schematic.

The output angle of the fiber optic angle sensor without nonlinear compensation is recorded as θ_1_, the output angle of the fiber optic angle sensor after MEA-BP compensation is θ_2_, and the angle value detected by the Hall angle sensor is θ. Ten groups of measurements were randomly selected for comparative analysis, as shown in Table 6.

**Table 6:** The measured data of three Angle measurement methods.

Uncompensated angle θ_1_	Angle after compensation θ_2_	Hall angle sensor θ
2.2	5.4	7.6
7.3	10.6	11.5
31.0	26.2	26.3
33.0	34.1	34.8
39.6	39.3	40.1
56.7	58.1	58.3
63.9	65.4	66.0
73.3	73.3	73.5
82.0	81.7	81.0
87.4	87.3	87.6

The smaller the mean square error of the experimental data, the higher the accuracy. Therefore, this paper uses the mean square error to reflect the accuracy of the detection results. Using the high-precision Hall angle sensor measurements as the reference values, the mean square errors *MSE*_1_ and *MSE*_2_ of the outputs without nonlinear compensation and after MEA-BP compensation are calculated as follows:

14 $$MS{E_1}=\frac{1}{{10}}\sum\limits_{{i=1}}^{{10}} {{{({\theta _1} - \theta )}^2}} ={\mathrm{8}}{\mathrm{.043}}$$

15 $$MS{E_2}=\frac{1}{{10}}\sum\limits_{{i=1}}^{{10}} {{{({\theta _2} - \theta )}^2}} ={\mathrm{0}}{\mathrm{.781}}$$

The results show that the accuracy of the fiber angle sensor is improved from 8.043 to 0.781 after nonlinear compensation by MEA-BP model.root mean square error(RMSE) From 2.836 to 0.884.

To evaluate the static repeatability of the fiber optic angle sensor, 10 static measurements were performed at three positions (0°,45°, and 80°) to assess its consistency, as shown in Table 7.

**Table 7:** Static repeatability of fiber optic angle sensor.

Angle	Number of tests	Maximum offset	Mean	Standard deviation
0°	10	0.2	0.12	0.07
45°	10	0.3	44.99	0.09
80°	10	0.2	80.07	0.07

In practical high-voltage disconnector monitoring systems, angular detection typically requires an absolute measurement error within ± 0.5° to ensure reliable judgment of opening and closing states. To further evaluate the performance of the compensation model from a reliability perspective, an error tolerance-based assessment method is introduced.Therefore, in this study, a tolerance threshold δ = 0.5° is adopted as the engineering evaluation criterion. For a prediction result, if its absolute error |θ_pred- θ_true| <δ, it is considered a “reliable prediction”. On the same test dataset, statistical analysis of the prediction results from the traditional BP compensation model and the MEA-BP compensation model is conducted, with the results shown in Table 8.

**Table 8:** Comparison of model prediction reliability based on error tolerance (δ = 0.5).

Evaluation metrics	Definition	traditional BP network	MEA-BP network
Precision	True reliable predictions / Model-judged reliable predictions	86.4%	96.2%
Recall	True reliable predictions / Actual need-reliable predictions	85.6%	95.4%
F1 score	2 × Precision × Recall / (Precision + Recall)	85.1%	94.7%
number of samples for reliable prediction	Number of samples with absolute error < 0.5°	491	491
Total test sample count		500	500

As shown in Table 8, under identical strict tolerance (δ = 0.5), the MEA-BP model achieves a significantly higher prediction reliability (96.2%) than the conventional BP network (86.4%). This indicates that the optimized MEA model’s output is highly likely to fall within a minimal neighborhood of values closely approximating the true value, demonstrating exceptional stability and accuracy. This finding, from the perspective of “prediction reliability,” complements and reinforces conclusions derived from earlier metrics such as MSE and linear error, collectively validating the MEA-BP model’s comprehensive superiority in enhancing sensor output performance.

## Summary

Based on the opening and closing characteristics and the working environment of high-voltage isolator, a linear-curve optical fiber angle sensor is designed to solve the nonlinear problems caused by the defects of mechanical sensor processing, temperature and humidity, and external light. The MEA-BP model is established to compensate the nonlinearity of the sensor.

(1) The idea of using optical fiber displacement sensor to convert the rotation angle of the isolator’s moving contact into measured displacement can avoid the influence of electromagnetic radiation around the isolator on the output value of the sensor.

(2) The nonlinear compensation fiber optic angle sensor and the high-precision Hall angle sensor were installed on the SBE27.5 kV / 2000 A isolator for experimental comparison. According to the measurement values of the Hall angle sensor, the accuracy of the fiber optic angle sensor reached 0.781, indicating that this solution has high accuracy and strong feasibility, making it suitable for monitoring the opening and closing of high-voltage switch isolators.

(3) System Limitations This solution still faces practical challenges: its accuracy depends on the precision of mechanical transmission components during manufacturing and assembly, and prolonged wear may compromise stability. The MEA-BP compensation model developed is static, requiring enhanced adaptability to drastic or long-term environmental changes. Additionally, the integration between the system and on-site intelligent control units, as well as its long-term operational reliability, need further validation in actual station environments.Given mechanical wear and long-term environmental effects, it is recommended to establish periodic calibration plans during field deployment, with online self-check/self-calibration mechanisms being a key component of future system integration.

(4) Future Work Directions The subsequent research will focus on: (1) Optimizing sensor architecture to explore more reliable optical angle measurement solutions or precision transmission mechanisms; (2) Developing adaptive compensation algorithms with online learning capabilities to enhance model generalization; (3) Conducting environmental adaptability reinforcement tests and long-term grid-connected evaluations at substations to facilitate the engineering application of the technology.

## Data Availability

Data AvailabilityThe datasets used and/or analysed during the current study available from the corresponding author on reasonable request.

## References

[1] Chen, W. J. et al. Study on Influence of the Disconnector Characteristics on Very Fast Transient Overvoltages in 1100 kV Gas Insulated Switchgear. *IEEE Trans. Power Delivery*. **30** (4), 2037–2044 (2015).

[2] Liu, S. et al. New Strategy for Mechanical Fault Diagnosis of Catenary High Voltage Isolating Switch. *J. East. China Jiaotong Univ.* **36** (05), 136–142 (2019).

[3] Liu, Y, et al. Contact State Detection Technology of GIS Disconnect Switch Based on Vibration Principle. *High Voltage Engineering*. **45**(05), 1591–1599. (2019).

[4] Bozhong, W. et al. Review on Breaking-closing Position Monitoring Method for Intelligent Disconnecting Switches[C]. *IOP Conference Series: Earth and Environmental Science*. **223**(1), 012026. (IOP Publishing, 2019).

[5] Shao, J., Yan, Y. & Qi, D. Detection and Status Identification of Switch Equipment in Substations Based on Huff Forest. *Autom. Electr. Power Syst.* **40** (11), 115–120 (2016).

[6] Teng, Y. et al. A novel method to recognize the state of high-voltage isolating switch[J]. *IEEE Trans. Power Del.* **34**(4), 1350–1356 (2019).

[7] Shi, J. et al. Design and realization of high voltage disconnector condition monitoring system. *Journal of Physics: Conference Series*. **887**(1), 012011. (IOP Publishing, 2017).

[8] Warren-Smith, S. C. et al. Stability of grating-based optical fiber sensors at high temperature. *IEEE Sens. J.* **19** (8), 2978–2983 (2019).

[9] Hou, C. et al. Prediction of synchronous closing time of permanent magnetic actuator for vacuum circuit breaker based on PSO-BP. *IEEE Trans. Dielectr. Electr. Insul.* **24** (6), 3321–3326 (2017).

[10] Guo, N. et al. Research on SOC fuzzy weighted algorithm based on GA-BP neural network and ampere integral method. *J. Eng.* **2019** (15), 576–580 (2019).

[11] Zhang, J. et al. An Efficient Optimization Algorithm for Extreme Value of Nonlinear Function Based on the SAGA and BP Algorithm. *IEEE Access.* **7**, 133058–133068 (2019).

[12] Zhang, J. et al. An Efficient Optimization Algorithm for Extreme Value of Nonlinear Function Based on the SAGA and BP Algorithm. *IEEE Access.* **7**, 133058–133068 (2019).

[13] Gaohui, F. et al. Research on Corona Current Mathematical Model Based on Neural Network Curve Fitting. *High. Voltage Eng.* **41** (03), 1034–1041 (2015).

[14] Wang, W. et al. A BP neural network model optimized by Mind Evolutionary Algorithm for predicting the ocean wave heights. *Ocean Eng.* **162**, 98–107 (2018).

[15] Luya, Y. & ZHANG, Y. Image enhancementbased detection method of non-soluble deposit densitylevelsofporcelaininsulators. *AutomationofElectric Power Syst.* **42** (14), 151–157 (2018).

[16] Cui, J. T. J. Z. & Zhuorang, C. J. Tang Junxiang,Zhang Zhuorang, et al. Research on Fast Fault Classification Method of Aero-Generator Rotary Rectifier Based on Extreme Learning Machine. *Proceedings of the CSEE*. **38**(08), 2458–2466 +2555 (2018).

[17] Li, G. & Li, W. Face feature point tracking based on mind evolutionary algorithm. *J. Jilin University(Engineering Technol. Edition)*, **45**(02), 606–612 (2015).

[18] Xu, L., Du, X. & Wang, B. Short-term traffic flow prediction model of wavelet neural network based on mind evolutionary algorithm. *Int. J. Pattern recognit. Artif. Intell.* **32** (12), 1850041 (2018).

[19] Hossain Lipu, M. S. et al. Optimal BP neural network algorithm for state of charge estimation of lithium-ion battery using PSO with PCA feature selection. *J. Renew. Sustain. Energy*. **9** (6), 064102 (2017).

[20] Heravi, A. R. & Hodtani, G. A. A new correntropy-based conjugate gradient backpropagation algorithm for improving training in neural networks. *IEEE Trans. neural networks Learn. Syst.* **29** (12), 6252–6263 (2018). 10.1109/TNNLS.2018.2827778 29993752

[21] Liang, Z. et al. Nonlinearity compensation of magneto-optic fiber current sensors based on WOA-BP neural network. *IEEE Sens. J.* **22** (20), 19378–19383 (2022).

[22] Mitrović, Z. et al. Improved method for calibration and nonlinearity correction of microwave power sensor. *Tehnički Vjesn.* **29** (2), 415–427 (2022).

[23] Jung, H. et al. Nano-Cracked Strain Sensor with High Sensitivity and Linearity by Controlling the Crack Arrangement. *Sensors* **19** (12), 2834 (2019). 31242680 10.3390/s19122834 PMC6631595

[24] Song, X., Fang, J. & Han, B. High-precision rotor position detection for high-speed surface PMSM drive based on linear Hall-effect sensors. *IEEE Trans. Power Electron.* **31** (7), 4720–4731 (2015).

